# Effects of drinking hydrogen-rich water on the quality of life of patients treated with radiotherapy for liver tumors

**DOI:** 10.1186/2045-9912-1-11

**Published:** 2011-06-07

**Authors:** Ki-Mun Kang, Young-Nam Kang, Ihil-Bong Choi, Yeunhwa Gu, Tomohiro Kawamura, Yoshiya Toyoda, Atsunori Nakao

**Affiliations:** 1 Department of Therapeutic Radiology, Gyeongsang National University Hospital, Gyeongsang Institute of Health Sciences, Jinju, Korea; 2 Department of Radiation Oncology, Catholic University Medical College, Seoul, Korea; 3 Graduate School of Health Science, Suzuka University of Medical Science, Suzuka, Japan; 4 Department of Cardiothoracic Surgery, University of Pittsburgh, Pittsburgh, Pennsylvania, USA; 5 Department of Surgery, University of Pittsburgh, Pittsburgh, Pennsylvania, USA

## Abstract

**Background:**

Cancer patients receiving radiotherapy often experience fatigue and impaired quality of life (QOL). Many side effects of radiotherapy are believed to be associated with increased oxidative stress and inflammation due to the generation of reactive oxygen species during radiotherapy. Hydrogen can be administered as a therapeutic medical gas, has antioxidant properties, and reduces inflammation in tissues. This study examined whether hydrogen treatment, in the form of hydrogen-supplemented water, improved QOL in patients receiving radiotherapy.

**Methods:**

A randomized, placebo-controlled study was performed to evaluate the effects of drinking hydrogen-rich water on 49 patients receiving radiotherapy for malignant liver tumors. Hydrogen-rich water was produced by placing a metallic magnesium stick into drinking water (final hydrogen concentration; 0.55~0.65 mM). The Korean version of the European Organization for Research and Treatment of Cancer's QLQ-C30 instrument was used to evaluate global health status and QOL. The concentration of derivatives of reactive oxidative metabolites and biological antioxidant power in the peripheral blood were assessed.

**Results:**

The consumption of hydrogen-rich water for 6 weeks reduced reactive oxygen metabolites in the blood and maintained blood oxidation potential. QOL scores during radiotherapy were significantly improved in patients treated with hydrogen-rich water compared to patients receiving placebo water. There was no difference in tumor response to radiotherapy between the two groups.

**Conclusions:**

Daily consumption of hydrogen-rich water is a potentially novel, therapeutic strategy for improving QOL after radiation exposure. Consumption of hydrogen-rich water reduces the biological reaction to radiation-induced oxidative stress without compromising anti-tumor effects.

## Background

Radiotherapy is one of the major treatment options for malignant neoplasms. Nearly half of all newly diagnosed cancer patients will receive radiotherapy at some point during treatment and up to 25% may receive radiotherapy a second time [1]. While radiotherapy destroys malignant cells, it adversely affects the surrounding normal cells [2]. Acute radiation-associated side effects include fatigue, nausea, diarrhea, dry mouth, loss of appetite, hair loss, sore skin, and depression. Radiation increases the long-term risk of cancer, central nervous system disorders, cardiovascular disease, and cataracts. The likelihood of radiation-induced complications is related to the volume of the irradiated organ, the radiation dose delivered, the fractionation of the delivered dose, the delivery of radiation modifiers, and individual radiosensitivity [3]. Most radiation-induced symptoms are believed to be associated with increased oxidative stress and inflammation, due to the generation of reactive oxygen species (ROS) during radiotherapy, and may significantly affect the patient's quality of life (QOL) [2].

Hydrogen, a therapeutic medical gas, has antioxidant properties and reduces inflammatory events in tissues [4-6]. Drinking liquids supplemented with hydrogen represents a novel method of hydrogen gas delivery that is easily translatable into clinical practice, with beneficial effects for several medical conditions, including atherosclerosis, type 2 diabetes, metabolic syndrome, and cognitive impairment during aging and in Parkinson's disease [7-11]. Currently, there is no definitive therapy to improve the QOL of patients receiving radiotherapy. Drinking solubilized hydrogen on a daily basis may be beneficial and would be quite easy to administer without complicating or changing a patient's lifestyle. We hypothesized that oral intake of hydrogen-rich water, generated *via *a magnesium stick, would reduce adverse events in patients receiving radiotherapy.

## Methods

### Subjects and design

The study was a two-arm, randomized, controlled clinical trial. Patients were randomly assigned to receive either hydrogen-rich water or placebo water on the first day of radiation treatment, and received follow-up questionnaires on compliance and potential adverse effects. Eligible patients were informed of the study during scheduling of pre-radiation testing. Patient characteristics, including tumor origin and the specifics of radiotherapy, are listed in Table 1. Forty-nine subjects (33 men and 16 women) were enrolled between April and October 2006. The age of the patients ranged from 21 to 82 years (mean age 58.6 years). All patients were diagnosed either histologically or pathologically with hepatocellular carcinoma (HCC) or metastatic hepatic tumors. All participants received 5040-6500 cGy of radiotherapy for 7-8 weeks using a 6 MV system (Cyber Knife, Fanuc, Yamanashi, Japan). The planned target volume of the initial field was assessed by a localization/simulation procedure or by computed tomography (CT)-assisted planning and encompassed the primary tumors and a 2 cm margin. Blocks were used to shield normal tissue.

**Table 1:** Patient Characteristics

	**water**	**Age**	**gender**	**times**	**diagnosis**	**isodose curve (%)**	**total cGy**	**volume (cc)**	**collimater (cc)**	**response**		**water**	**age**	**gender**	**times**	**diagnosis**	**isodose curve (%)**	**total cGy**	**volume (cc)**	**collimater (cc)**	**response**
		
1	placebo	76	M	3 3	HCC	80 75	3,900 3,900	2.521 2.746	7.5 7.5	NR		HW	52	M	3	liver meta of colon ca	74	3,600	12.283	15	NR
		
2	placebo	82	M	1	HCC	70	1,200	11.769	20	CR		HW	56	M	3	liver meta of colon ca	85	3,600	2.552	12.5	PR
		
3	placebo	57	F	3	bile duct ca	80	3,000	40.334	30	PR		HW	77	F	3	liver meta of colon ca	75	3,000	107.136	20	CR
		
4	placebo	47	F	9	liver meta. of sarcoma	80 82 84	3,600 3,600 3,900	10.628 6.542 2.673	25 20 15	NR		HW	57	M	3	HCC	70	3,600	47.679	15	NR
		
5	placebo	50	F	3	liver meta of colon ca	80	3,900	16.237	20	NR		HW	66	M	3	HCC	80	3,600	16.216	25	PR
		
6	placebo	21	F	3	liver meta. of ovarian ca	85	3,600	29.398	30	CR		HW	57	M	3	HCC	80	3,600	35.303	30	NR
		
7	placebo	65	M	3	liver meta. of rectal ca	70	3,000	182.871	40	PR		HW	47	M	3	HCC	77	3,000	17.65	20	CR
		
8	placebo	73	M	3	liver meta. of rectal ca	75	3,600	37.937	20	PR		HW	49	M	3	HCC	80	3,300	53.578	12.5	PR
		
9	placebo	58	M	3	liver meta. of pancreatic ca	75	3,000	65.637	35	CR		HW	71	F	3	HCC	85	3,000	3.861	10	NR
		
10	placebo	64	M	3	HCC	70	3,000	140.136	20	PR		HW	45	M	3	HCC	80	3,600	28.286	15	NR
		
11	placebo	65	F	3	HCC	70	3,600	48.645	25	PR		HW	45	F	3	liver meta. of gastric ca	85	3,000	38.938	15	PR
		
12	placebo	80	M	3	HCC	80	3,000	209.954	25	NR		HW	56	F	3	Adrenal metastasis of HCC	80	3,600	9.494	15	PR
		
13	placebo	56	M	3	HCC	85	3,600	15.365	15	CR		HW	49	M	3	Adrenal metastasis of HCC	75	3,000	91.223	20	NR
		
14	placebo	61	F	3	HCC	70	3,000	98.957	30	NR		HW	60	M	3	LN metastasis of HCC	75	3,000	120.366	25	NR
		
15	placebo	46	M	3	HCC	80	3,000	20.848	25	CR		HW	47	M	3	LN metastasis of HCC	80	3,000	80.459	25	NR
		
16	placebo	70	F	3	HCC	85	3,600	16.908	20	PR		HW	50	M	3	HCC	75	3,600	29.422	20	NR
		
17	placebo	44	M	3	HCC	85	3,600	16.612	30	NR		HW	49	F	3	HCC	70	3,000	156.289	40	PR
		
18	placebo	48	M	3	HCC	85	3,000	35.093	20	NR		HW	63	F	3	HCC	75	3,900	5.425	20	NR
		
19	placebo	76	F	3	HCC	85	3,600	5.75	15	NR		HW	51	M	3	HCC	70	4,000	28.637	35	NR
		
20	placebo	60	M	3	HCC	83	3,600	6.802	12.5	NR		HW	67	F	3	HCC	80	3,600	20.122	20	PR
		
21	placebo	77	M	3	HCC	75	3,300	33.282	25	PR		HW	56	M	3	HCC	70	3,600	23.5	20	CR
		
22	placebo	55	M	3	HCC	83	3,600	11.963	20	NR		HW	78	F	3	HCC	83	3,600	26.456	25	NR
		
23	placebo	57	M	3	HCC	70	3,000	75.782	40	NR		HW	56	M	3	HCC	77	3,600	31.908	20	CR
		
24	placebo	65	M	2	HCC	75	3,000	55.191	25	NR		HW	60	M	3	HCC	70	3,600	36.479	30	PR
		
												HW	70	M	3	HCC	76	3,600	63.434	40	NR

M: male, F: female, HCC: hepatocellular carcinoma, NR: no response, PR: partial response, CR: complete response, HW: hydrogen water

Hydrogen-rich water was produced by placing a metallic magnesium stick (Doctor SUISOSUI^®^, Friendear, Tokyo, Japan) into drinking water (Mg + 2H_2_O → Mg (OH)_2 _+ H_2_; final hydrogen concentration: 0.55~0.65 mM). The magnesium stick contained 99.9% pure metallic magnesium and natural stones in a polypropylene and ceramic container. The subjects were randomly assigned to groups to either drink hydrogen-rich water for 6 weeks (n = 25) or drink water containing a placebo (a casing-only stick placed in drinking water) (n = 24). Subjects were provided with four 500 mL bottles of drinking water per day and instructed to place two magnesium sticks in each bottle of water at the end of each day in preparation for consumption the following day. Participants were asked to drink 200-300 mL from one bottle each morning, and 100-200 mL every a few hours from the remaining three bottles. Subjects were instructed to reuse the magnesium sticks by transferring the sticks to a new bottle of water after use. The subjects were expected to consume 100-300 mL of hydrogen-rich water more than 10 times per day for a total minimum consumption of 1500 mL (1.5 L) and a maximum consumption of 2000 mL (2.0 L). Oral intake of hydrogen water or placebo water started on the first day of radiotherapy and continued for 6 weeks. All the patients survived through the 6 week follow-up period when the QOL questionnaire was administered. This study was conducted in accordance with Good Clinical Practice guidelines and the ethical principles of the Declaration of Helsinki (2000). The study protocol and materials were approved by the Institutional Review Board of Catholic University Medical College, and all subjects provided written informed consent prior to participation.

### QOL Assessment

The Korean version of the European Organization for Research and Treatment of Cancer's QLQ-C30 instrument with modifications was used to evaluate global health status and create QOL scales [12]. The descriptive, mailed survey developed by our institute was used in this study. The questionnaire contains five functional scales (physical, cognitive, emotional, social, and role-functioning), three symptom scales (pain, fatigue, and nausea/vomiting), and six single items to assess additional symptoms (dyspnea, insomnia, loss of appetite, constipation, diarrhea). For all items, a response scale ranging from 0-5 was used. A higher score reflected a higher level of symptoms and decreased QOL. Assessments were performed before radiotherapy and every week for 6 weeks after the initiation of radiotherapy.

### Biomarker analysis

The concentrations of derivatives of reactive oxidative metabolites (dROMs) and biological antioxidant power (BAP) in the peripheral blood were assessed using a free Radical Analytical System (FRAS4; H&D, Parma, Italy) on the first day of radiation therapy (week 0) and after 6 weeks of radiotherapy. Blood samples were obtained from all patients after overnight fasting. FRAS4 dROMs kits were used to measure total hydroperoxide levels, which are representative of the total dROMs produced as a result of peroxidation chain reactions of proteins, lipids, and amino acids. Results were expressed in U.CARR; 1 U.CARR is equivalent to 0.08 mg/dl of hydrogen peroxide and the value is directly proportional to the concentration, according to Lambert-Beer's law.

Redox potential, including glutathione peroxidase and superoxide dismutase, were determined using the FRAS4 BAP test [13]. Described briefly, the samples to be tested were dissolved in a colored solution containing a source of ferric ions and a chromogenic substance (a sulfur-derived compound). After a 5-minute incubation period, the degree of discoloration and intensity of the change were directly proportional to the ability of the plasma to reduce ferric ions. The amount of reduced ferric ions was calculated using a photometer to assess the intensity of discoloration; BAP results were expressed as µmol/l of reduced Fe/l.

Blood chemistry tests for aspartate aminotransferase, alanine aminotransferase, gamma-glutamyl transpeptidase (γ-GTP), and total cholesterol, as well as blood hematology tests for red blood cell count, white blood cell count, and platelet count were conducted at week 0 and week 6 using standard assays in an accredited hospital laboratory.

### Assessment of response

Patients underwent dynamic CT scans 1-2 months after completion of radiation treatment and tumor response was checked at 2-3 month intervals thereafter. Treatment response and local recurrence were evaluated using follow-up dynamic CT scans and serum tests for alpha-fetoprotein (AFP) and prothrombin, which is induced by vitamin K absence or antagonist-II (PIVKA-II). Tumor response was determined by the criteria established by Kwon *et al. *[14]. Described briefly, complete response (CR) was defined as the disappearance of any intratumoral arterial enhancement in all target lesions. Partial response (PR) was defined as at least a 30% decrease in the sum of the diameters of viable target lesions. Progressive disease (PD) was defined as an at least 20% increase in the sum of the diameters of viable target lesions or the appearance of a new lesion. Stable disease (SD) was defined as a tumor status that did not meet any of the above criteria.

### Statistical analysis

Unpaired *t *tests were used to compare numerical data and the Yates 2 x 2 chi-square test or Fisher exact probability test was used to compare categorical data. Statistical analyses were performed using SAS 6.13 software (SAS Institute Inc., Cary, NC). The sample size of 49 patients was sufficient to detect a change in mean scores of RORTC QLQ-C30.

## Results

### Hydrogen water improved the QOL of patients receiving radiotherapy

**Figure 1:**
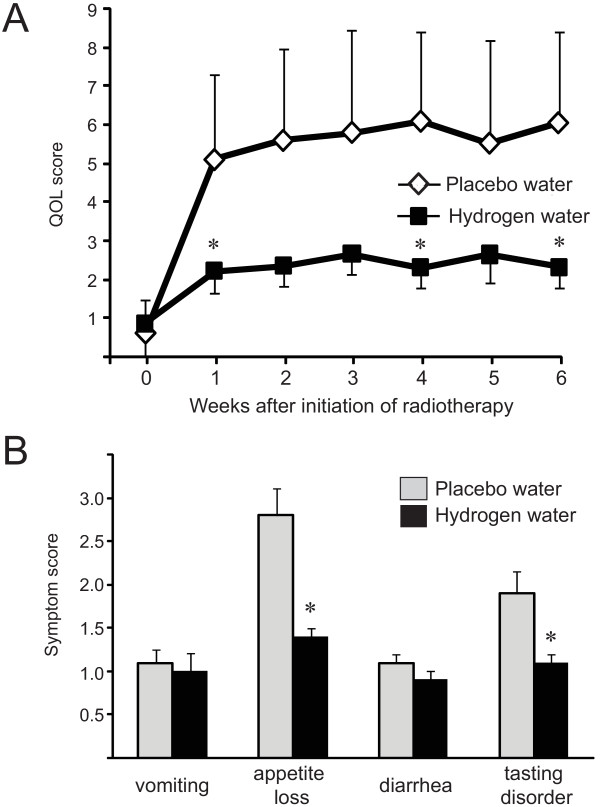
**Placebo water and hydrogen water improved the QOL of patients receiving radiotherapy**. A. Weekly assessment of the patients' QOL. B. Scoring system of GI symptoms after 6 weeks of radiotherapy with or without hydrogen water.

The QOL of the patients who were given placebo water deteriorated significantly within the first month of radiotherapy (Figure 1A). There were no differences between the groups in the QOL subscales for fatigue, depression, or sleep. Gastrointestinal (GI) symptoms are one of the most common complaints of patients undergoing radiotherapy and are considered to have a high impact on the patient's QOL after 6 weeks of radiotherapy. The patients consuming hydrogen water experienced significantly less appetite loss and fewer tasting disorders compared to the patients consuming placebo water. No significant difference was seen in the mean scores for vomiting or diarrhea (Figure 1B).

### Hydrogen water mitigated oxidative stress marker during radiotherapy

**Figure 2:**
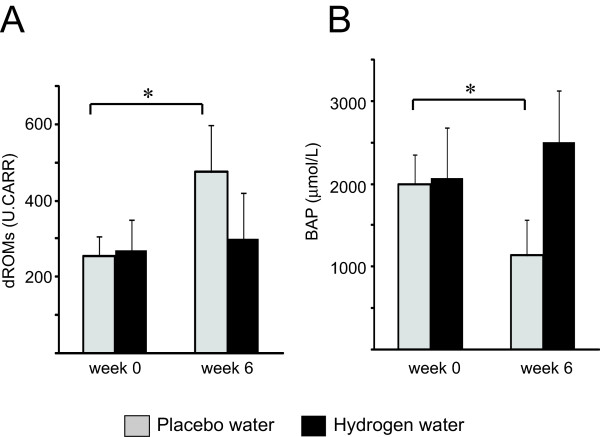
**Hydrogen water mitigated oxidative stress marker during radiotherapy**. Antioxidative effects in patients with placebo water (n = 24) and hydrogen rich water (n = 25). The dROM level (A) represents the total level of peroxide metabolities, and BAP (B) reflects serum antioxidant capacity.

Before treatment, there were no differences in total hydroperoxide levels, representative of total dROM levels, between the treatment groups. Radiotherapy markedly increased total hydroperoxide levels in the patients consuming placebo water. However, drinking hydrogen water prevented this increase in total serum hydroperoxide, as determined by the dROM test (Figure 2A), indicating decreased oxidative stress during radiotherapy in the patients who consumed hydrogen water. Similarly, endogenous serum antioxidant activity significantly deteriorated during radiotherapy in the patients consuming placebo water, and biologic antioxidant activity was maintained in patients who consumed hydrogen-rich water, even after 6 weeks of radiotherapy (Figure 2B).

### Hydrogen water did not compromise the radiation treatment efficacies

Tumor response to radiotherapy was similar between the treatment groups, and 12 of 24 (50.0%) patients in the placebo group and 12 of 25 (48%) patients in hydrogen water group exhibited either a completed response (CR) or a partial response (PR). There were no patients in either group with progressive disease (PD) during the follow-up period (3 months). Thus, drinking hydrogen water did not compromise the anti-tumor effects of radiotherapy.

### Hydrogen treatment did not alter liver function or blood composition during radiotherapy

There were no significant differences in aspartate aminotransferase, alanine aminotransferase, gamma-glutamyl transpeptidase (γ-GTP) and total cholesterol levels at week 0 and week 6, regardless of the type of water consumed (Table 2), indicating that hydrogen water consumption did not alter liver function. Similarly, there were no significant differences in red blood cell count, white blood cell count, or platelet count between patients consuming hydrogen water and patients consuming placebo water (Table 3).

**Table 2:** Changes in liver function tests

	Placebo			Hydrogen water		
	**all (n = 25)**	**male (n = 17)**	**female (n = 8)**	**all (n = 25)**	**male (n = 16)**	**female (n = 9)**

AST(IU/L)						
Week 0	24.8 ± 9.1	25.6 ± 5.7	23.1 ± 10.4	25.3 ± 6.7	25.9 ± 5.3	23.9 ± 8.3
Week 6	26.3 ± 6.7	26.9 ± 7.1	25.4 ± 6.8	26.8 ± 8.2	27.2 ± 9.9	26.4 ± 5.1

ALT(IU/L)						
Week 0	27.4 ± 15	28.1 ± 11	26.5 ± 17	26.9 ± 8.7	27.1 ± 6.7	26.7 ± 10.3
Week 6	28.8 ± 14	28.7 ± 16	27.6 ± 12	28.1 ± 6.5	28.8 ± 7.3	27.6 ± 9.9

γ-GPT(IU/L)						
Week 0	61.9 ± 54.3	62.3 ± 35.6	60.5 ± 64.7	62.3 ± 26.2	62.1 ± 34.8	62.4 ± 47.9
Week 6	62.8 ± 22.8	63.2 ± 16.5	62.7 ± 25.9	63.6 ± 36.2	63.9 ± 54.2	63.2 ± 27.4

AST(IU/L)						
Week 0	24.8 ± 9.1	25.6 ± 5.7	23.1 ± 10.4	25.3 ± 6.7	25.9 ± 5.3	23.9 ± 8.3
Week 6	26.3 ± 6.7	26.9 ± 7.1	25.4 ± 6.8	26.8 ± 8.2	27.2 ± 9.9	26.4 ± 5.1

**Table 3:** Peripheral blood cell counts

	Placebo			Hydrogen water		
	**all (n = 25)**	**male (n = 17)**	**female (n = 8)**	**all (n = 25)**	**male (n = 16)**	**female (n = 9)**

The number of leukocytes (× 10^2 ^/μL)						
Week 0	55.8 ± 15.6	58.5 ± 12.7	52.8 ± 16.4	56.2 ± 16.7	57.3 ± 17.2	55.4 ± 15.1
Week 6	53.9 ± 21.4	54.1 ± 22.7	53.7 ± 19.8	54.7 ± 28.7	55.1 ± 31.2	53.8 ± 19.4

The number of erythrocytes (× 10^4 ^/μL)						
Week 0	474.2 ± 38.3	492.3 ± 45.8	460.8 ± 30.5	482.5 ± 42.1	496.6 ± 50.7	472.9 ± 36.4
Week 6	462.1 ± 52.4	473.8 ± 42.1	456.4 ± 62.2	479.5 ± 36.5	486.4 ± 29.4	470.7 ± 40.5

The number of thrombocytes (×10^4 ^/μL)						
Week 0	25.7 ± 6.5	26.4 ± 4.7	24.7 ± 5.9	26.4 ± 7.1	26.9 ± 5.5	26.1 ± 4.8
Week 6	24.5 ± 4.7	25.9 ± 2.8	23.4 ± 6.4	25.7 ± 4.8	26.1 ± 4.7	25.3 ± 3.9

## Discussion

To our knowledge, this is the first report demonstrating the benefits of drinking hydrogen water in patients receiving radiation therapy for malignant tumors. This finding may provide the foundation for a clinically applicable, effective, and safe strategy for the delivery of hydrogen gas to mitigate radiation-induced cellular injury. Patients experience GI symptoms and decreased QOL during radiotherapy. These symptoms usually occur as a result of the body repairing damage to healthy cells, are particularly common towards the end of a course of radiation treatment, and can last for some time. The symptoms and their impact on QOL can be worsened by having to travel to the hospital each day. Drinking hydrogen-rich water improved the QOL of the patients receiving radiotherapy and did not require additional hospital visits. Although overall survival of patients with malignant tumors should remain oncologists' primary concern, survival should also be interpreted in light of symptom palliation and overall QOL, because the side effects of radiotherapy may negate the putative benefit of improved survival. Oral intake of daily hydrogen-supplemented water might be a prophylactic strategy to improve QOL of the patients receiving radiotherapy.

Although the mechanisms underlying the beneficial effects of hydrogen-rich water during radiotherapy have not been clearly elucidated, drinking hydrogen-supplemented water reduced dROM levels and maintained BAP levels in the serum, suggesting hydrogen-rich water exhibits potent systemic antioxidant activity. Previous experimental studies have linked daily consumption of hydrogen-rich water with improvement of a number of conditions in rodent models, including reducing atherosclerosis in apolipoprotein E knockout mice [10], alleviating cisplatin-induced nephrotoxicity [15], reducing vitamin C deficiency-induced brain injury [16], preventing chronic allograft nephropathy after renal transplantation [17], and ameliorating cognitive defects in senescence-accelerated mice [9] and a Parkinson's disease model [7]. In human studies, consumption of hydrogen-rich water prevented adult-onset diabetes and insulin resistance [11], as well as oxidative stress in potential metabolic syndrome [8].

Radiotherapy is associated with an increase in ROS, followed by damage to DNA, lipids, and proteins, and activation of transcription factors and signal transduction pathways. It has been estimated that 60-70% of the ionizing radiation-induced cellular damage is caused by hydroxyl radicals [18]. Therefore, a number of trials with the goal of reducing adverse effects due to excess ROS production have been performed with antioxidants delivered during the course of radiotherapy. Supplementation with α-tocopherol improves the salivary flow rate and maintains salivary parameters [19]. Treatment with the antioxidant enzyme superoxide dismutase prevented radiotherapy-induced cystitis and rectitis in bladder cancer patients receiving radiotherapy [20]. In addition, the combined use of pentoxifylline and vitamin E reduced radiation-induced lung fibrosis in patients with lung cancer receiving radiotherapy [21]. Thus, in general, supplementation with antioxidants is likely to offer overall benefits in the treatment of adverse effects of radiotherapy. However, not all antioxidants can afford radioprotection [22-24]. Furthermore, of significant concern is the finding that high doses of antioxidants administered as adjuvant therapy might compromise the efficacy of radiation treatment and increase of the risk of local recurrence of cancer [25,26]. Hence, the relatively lower toxicity associated with the use of these antioxidant agents is appealing, but not at the cost of poor tumor control. In contrast, in this study, drinking hydrogen-rich water did not affect radiotherapy's anti-tumor effects. Our results may suggest that hydrogen water functions not only as an antioxidant, but also plays a protective role by inducing radioprotective hormones or enzymes. Although further studies are warranted to elucidate the safety of hydrogen-rich water and determine the optimal concentration of hydrogen in drinking water, as well as involved mechanisms, daily intake of hydrogen-rich water may be a promising approach for counteracting radiation-induced impairments to QOL. This therapeutic use of hydrogen is also supported by the work of Qian *et al.*, who demonstrated that treating human lymphocyte AHH-1 cells with hydrogen before irradiation significantly inhibited ionizing irradiation-induced apoptosis and increased cell viability *in vitro*. They also showed that injection of hydrogen-rich saline could protect the gastrointestinal endothelia from radiation-induced injury, decrease plasma malondialdehyde and intestinal 8-hydroxydeoxyguanosine levels, and increase plasma endogenous antioxidants *in vivo *[27].

## Conclusions

In conclusion, our study demonstrated that drinking hydrogen-rich water improved QOL and reduced oxidative markers in patients receiving radiotherapy for liver tumors. This novel approach of oral intake of hydrogen-rich water may be applicable to a wide range of radiation-related adverse symptoms.

## List of abbreviations

ROS: reactive oxygen species; QOL: quality of life

## Competing interests

The authors declare that they have no competing interests.

## Authors' contributions

KMK, YNK and IBC participated in the radiation therapy and the data accumulation. YG participated in the design of the study and performed the statistical analysis. TK and YT and participated in its design and coordination. AN conceived of the study, and drafted the manuscript. All authors read and approved the final manuscript.
